# Supplementary material for: Resolving challenges in deep learning-based analyses of histopathological images using explanation methods
Source: Sci Rep. 2020 Apr 14;10:6423. doi: 10.1038/s41598-020-62724-2 (PMC7156509; doi:10.1038/s41598-020-62724-2)
Supplement: Supplementary file 1 — Supplementary Information. [file 41598_2020_62724_MOESM1_ESM.pdf]

Supplemental Document for
Resolving challenges in deep learning-based analyses of histopathological images using explanation methods

Miriam Hägele, Philipp Seegerer, Sebastian Lapuschkin, Michael Bockmayr,
Wojciech Samek, Frederick Klauschen, Klaus-Robert Müller and Alexander Binder

Appendix A
Confusion matrices

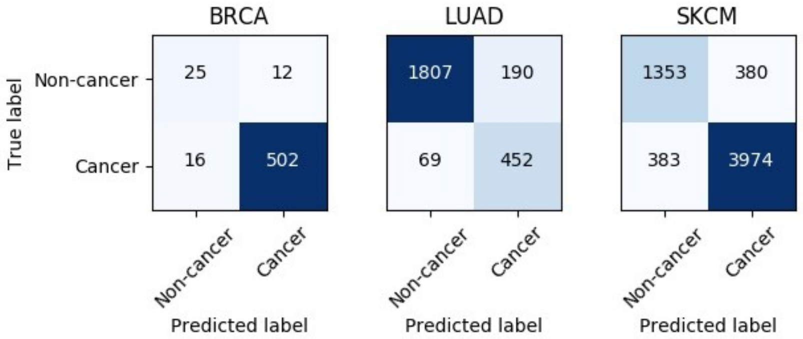

Fig. 1. Confusion matrices of the classifiers discriminating between cancerous and healthy tissue in the three studied tumor entities, namely invasive breast cancer (BRCA), lung adenocarcinoma (LUAD) and cutaeneous malignant melanoma (SKCM). When considering these results, please keep the different label distributions of the datasets in mind.

Appendix B
Detailed information on available annotations for evaluation on cell level

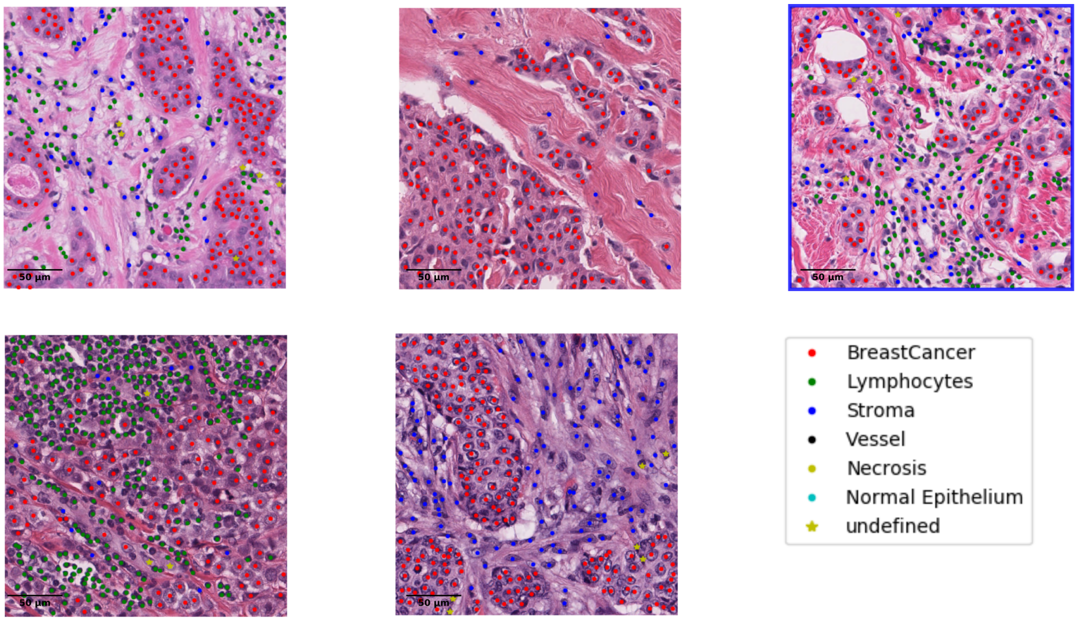

Fig. 2. Chosen exemplary sample tiles from the BRCA project, superimposed with the extensive single cell annotations. The blue box marks the example shown in the paper.

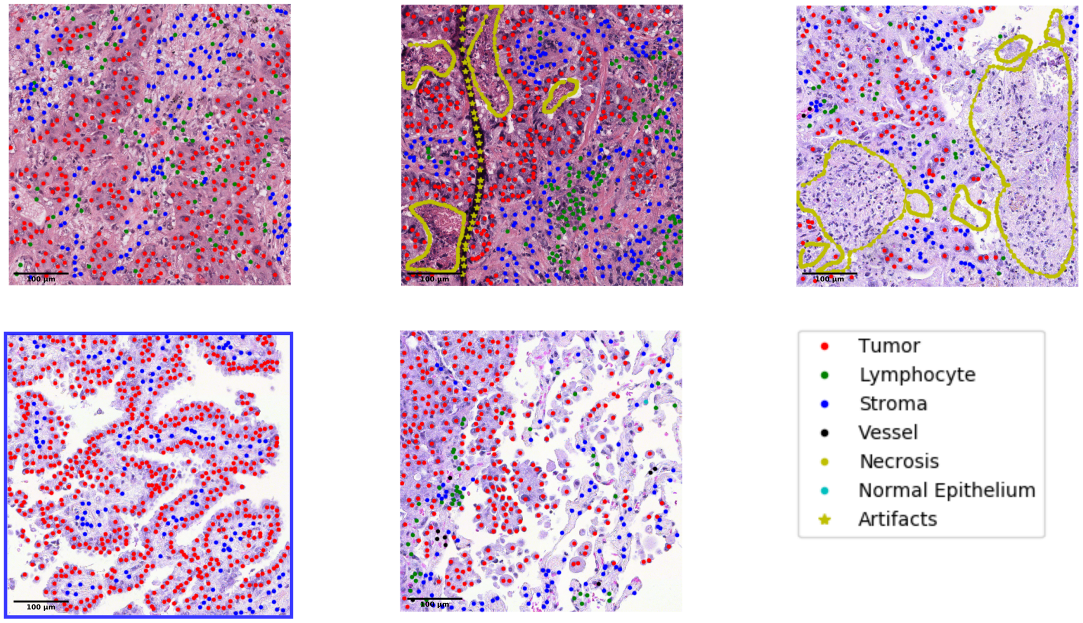

Fig. 3. Chosen exemplary sample tiles from the LUAD project, superimposed with the extensive single cell annotations. The blue box marks the example shown in the paper.

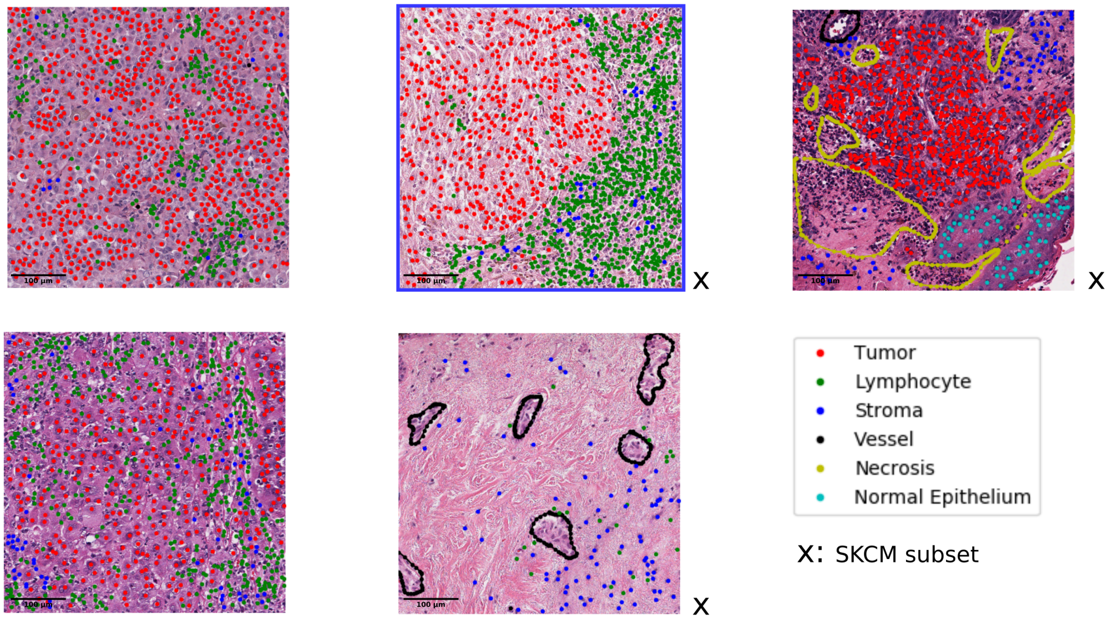

Fig. 4. Chosen exemplary sample tiles from the SKCM project, superimposed with the extensive single cell annotations. The blue box marks the example shown in the paper. The black marks show the subset which excludes the two tiles that contain tumour-infiltrating lymphocytes.

TABLE I
SUMMARY OF THE AVAILABLE ANNOTATIONS FOR EVALUATION ON CELL LEVEL.

Tumor entity	Total number of cells	Number of cancer cells
BRCA	1,803	820
SKCM	3,961	2,247
LUAD	2,722	1,650

Appendix C
Data sampling strategies

TABLE II
Performances of models with different sampling ratios.

Sampling ratio	F_1-score	Recall (*cancer* class)	Precision (*cancer* class)
0.5	0.92	0.88	0.97
0.8	0.93	0.92	0.95

Appendix D
Uncovering biases

I. Dataset bias

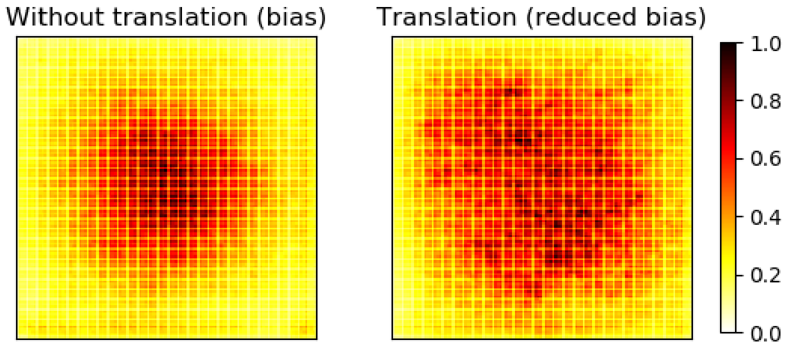

Fig. 5. Mean absolute relevances (of 896) patches of the "biased" (left) and the "unbiased" classifier (right). As expected relevance is predominately distributed in the center of the heatmap for the biased classifier.

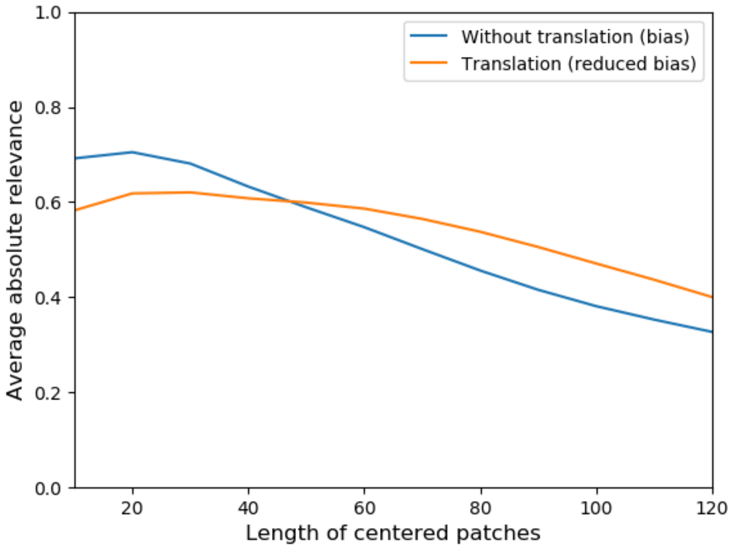

Fig. 6. Evaluation of the effect of training on center biased data. We compare the mean amount of absolute relevance as a function of relative, centered areas on an independent (not affected) test dataset.

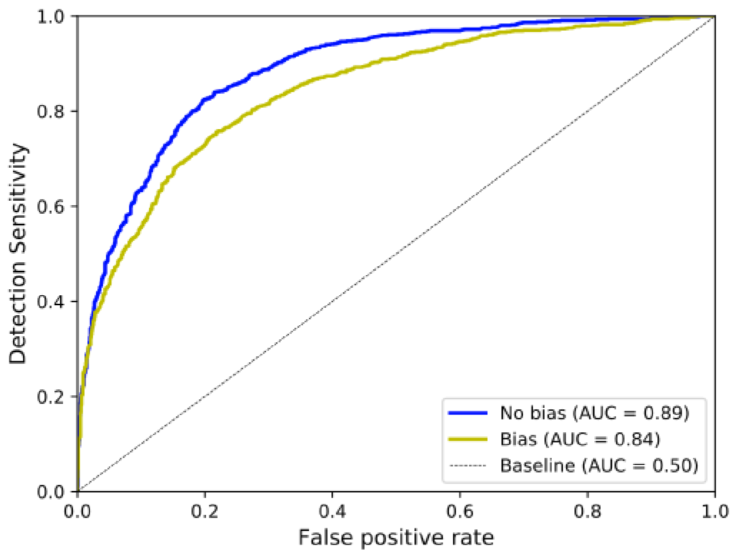

Fig. 7. Receiver operating characteristic (ROC) curve on seven held-out, extensively labelled 1000x1000px tiles of the corresponding dataset. The ROC curve improves when counteracting the bias by random translations.

II. Sampling bias

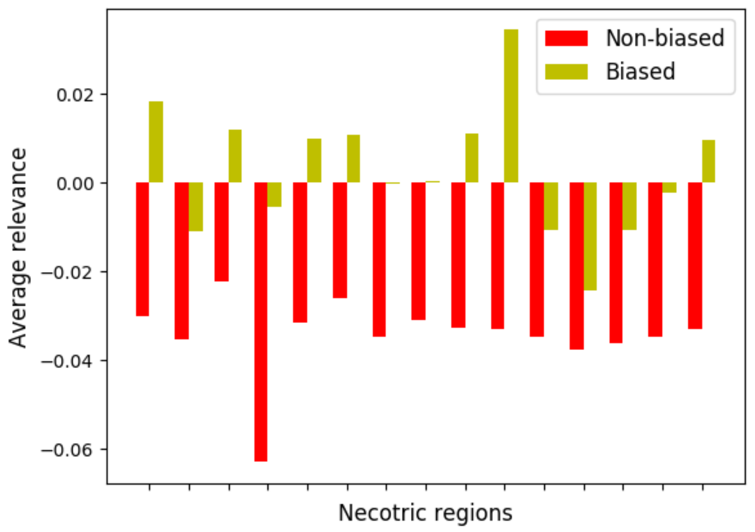

Fig. 8. Evaluation of all annotated necrosis regions on exemplary necroses tiles (cf. Fig. 9). Comparison between the average relevance per region depending on the classifier.

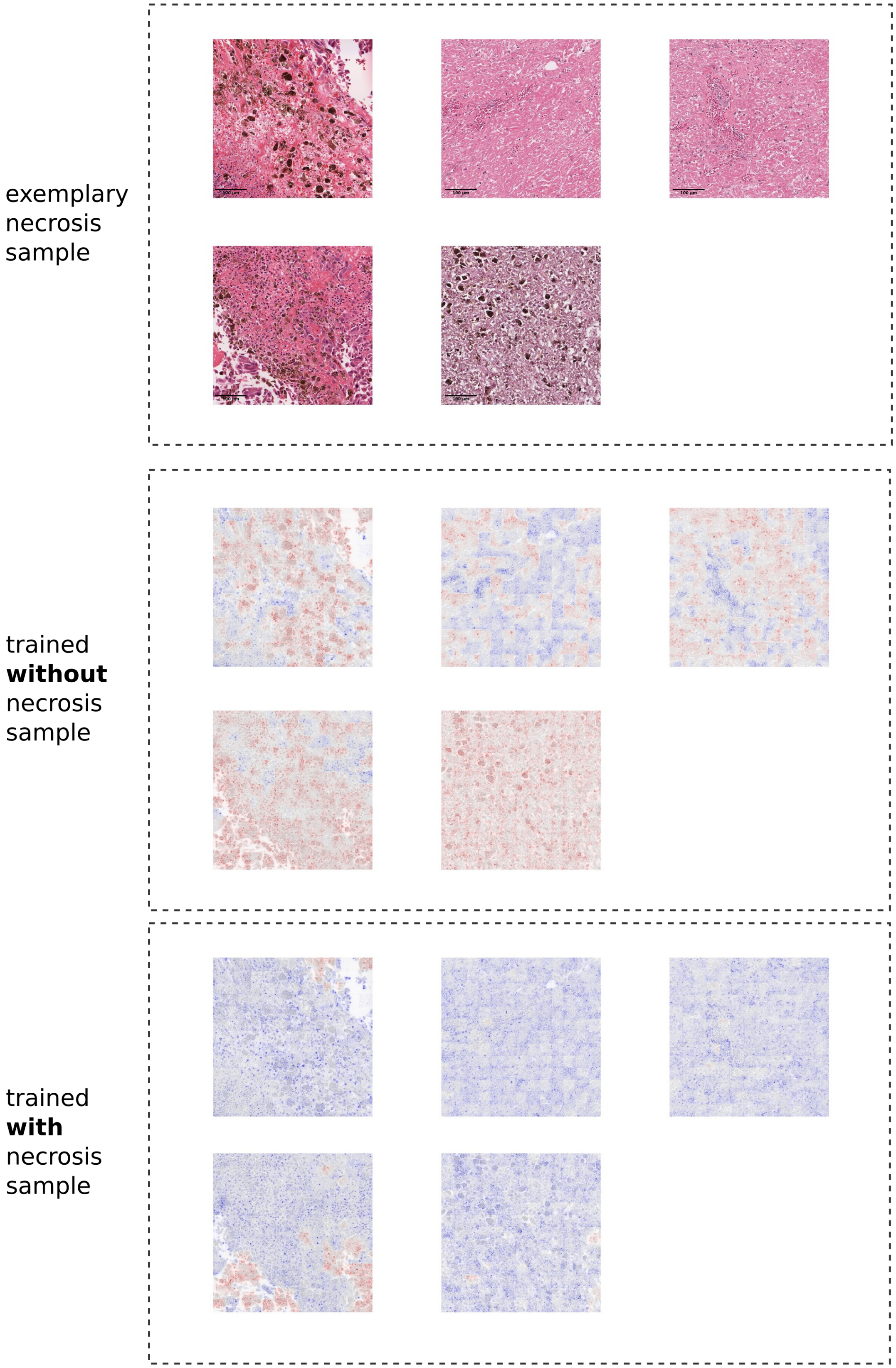

Fig. 9. Exemplary H&E tissue samples of necroses as well as heatmaps for both classifiers, i. e. trained on a comprehensive dataset and trained on a dataset lacking necroses samples respectively.

III. Class correlated bias

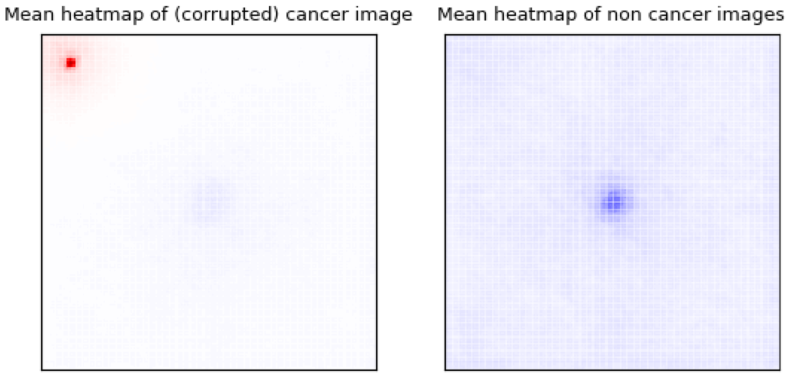

Fig. 10. Average heatmaps for class *cancer* of images classified as cancer (left) or classified as non-cancer (right).

Appendix E
ROC Curve for GradCam

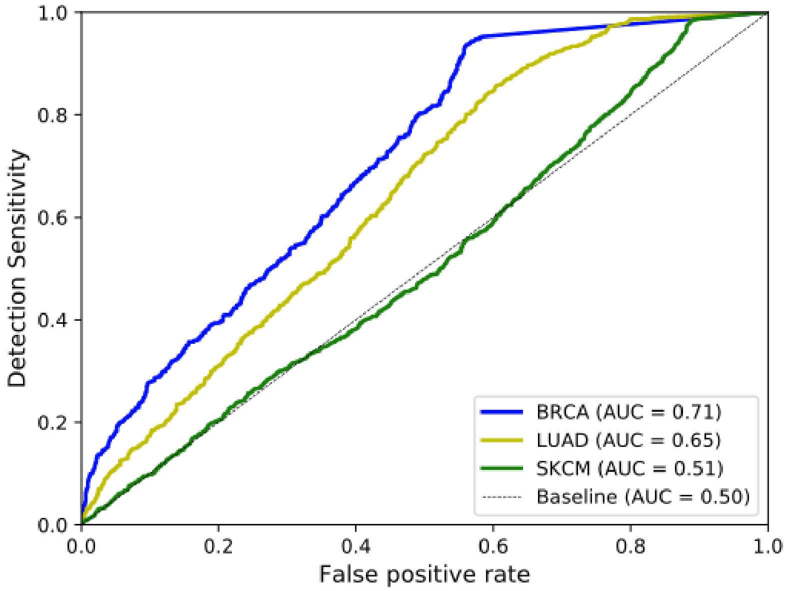

Fig. 11. Receiver operating characteristic (ROC) curves for GradCam heatmaps on all three studied tumor entities.
